# Supplementary material for: Endogenous IL-33 Accelerates Metacestode Growth during Late-Stage Alveolar Echinococcosis
Source: Microbiol Spectr. 2023 Feb 14;11(2):e04239-22. doi: 10.1128/spectrum.04239-22 (PMC10101030; doi:10.1128/spectrum.04239-22)
Supplement: Supplemental file 2 — Supplemental material. Download spectrum.04239-22-s0002.pdf, PDF file, 2.0 MB [file spectrum.04239-22-s0002.pdf]

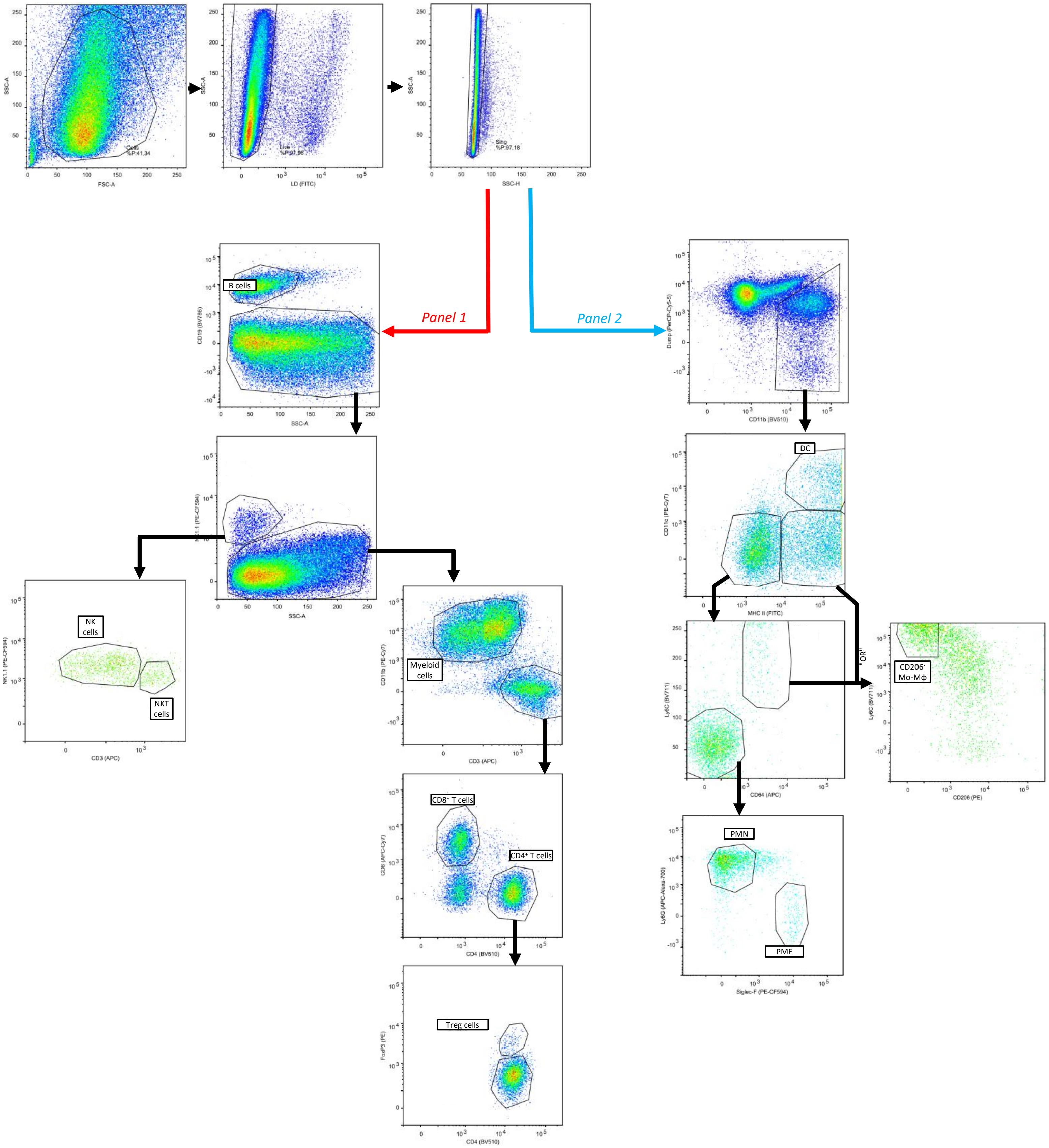

Supplementary Figure 1. Gating strategy. After an initial data cleaning (removing debris, dead cells and doublets), two different strategies were applied, depending on the antibodies panel. For Panel 1, B cells ($CD19^{+}$) were first isolated, then NK and NKT cells (SSC^{Lo} and $NK1.1^{+}$), which were then discriminated as being $CD3^{-}$ and $CD3^{+}$, respectively. Among $NK1.1^{-}$ cells, $CD11b^{+}CD3^{-}$ were identified as myeloid cells, and $CD11b^{-}CD3^{+}$ as T cells, further discriminated into $CD3^{+}CD8^{+}$ and $CD3^{+}CD4^{+}$, which included $CD4^{+}FoxP3^{+}$ "T_{reg}" cells. For Panel 2, myeloid cells were isolated as being $CD11b^{+}Dump^{-}$ (Dump: CD3, CD19, NK1.1). First, dendritic cells (DC) were identified as $MHC\text{-}II^{Hi}CD11c^{+}$ cells. Monocytes/macrophages were defined using an "OR" Boolean gate of $MHC\text{-}II^{+}CD11c^{-}$ cells and $MHC\text{-}II^{-}CD64^{+}Ly6C^{+}$ cells, then $CD206^{-}$ monocytes/macrophages were discriminated. Among the $MHC\text{-}II^{-}CD64^{-}Ly6C^{-}$ remaining cells, polymorphonuclear neutrophils (PMN) and polymorphonuclear eosinophils (PME) were defined as $Ly6G^{+}$ and $Siglec\text{-}F^{+}$, respectively.

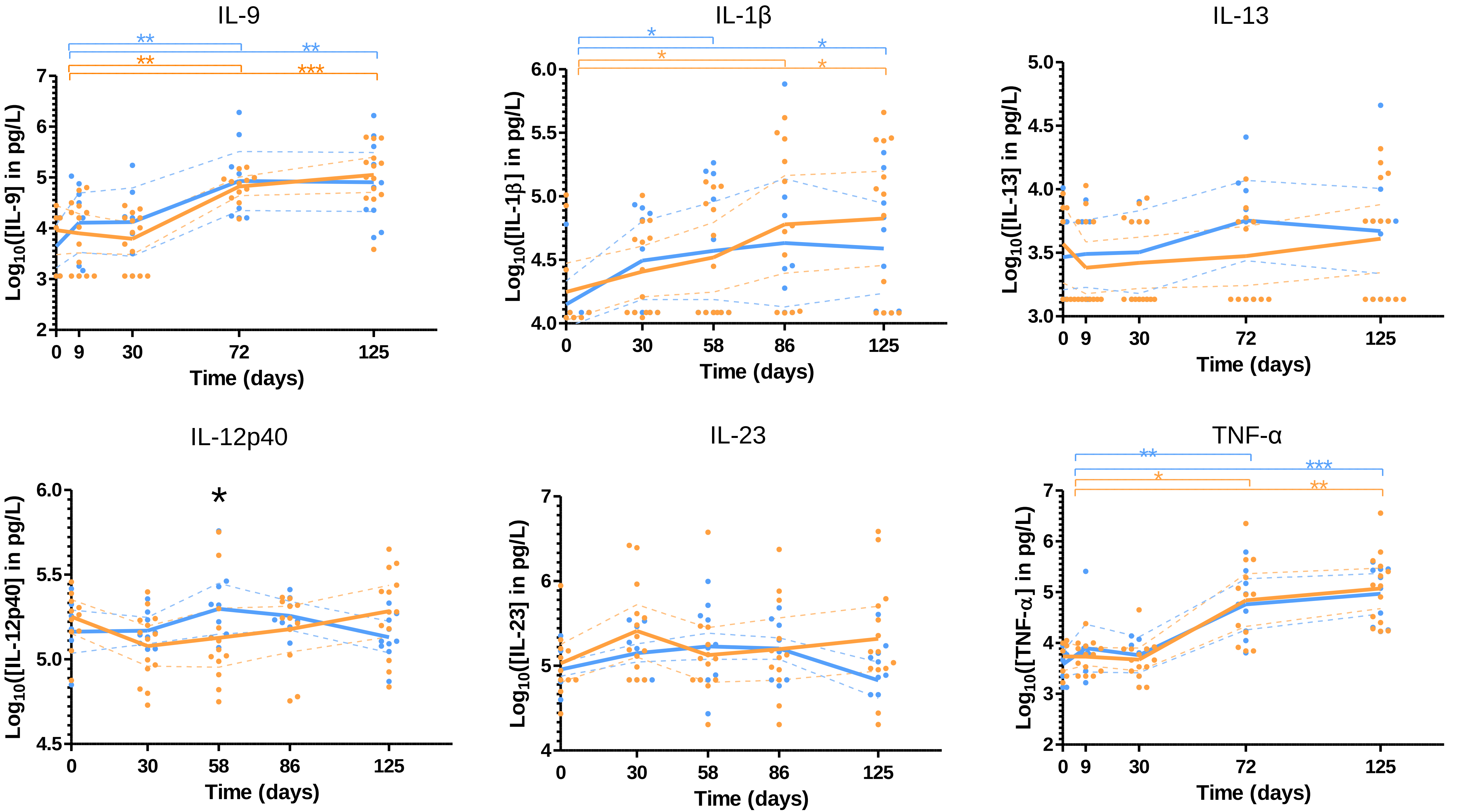

Supplementary Figure 2. Concentrations of cytokines in peritoneal lavages. Cytokines concentrations determined by flow cytometry in peritoneal lavages of WT and IL-33$^{-/-}$ at 4 months post infection. Data obtained from one experiment, with 5 uninfected, 6 WT and 6 IL-33$^{-/-}$ mice. Plots show mean ± SD. Significance was tested using the Kruskal-Wallis test with Dunn's multiple comparison *post-hoc* analysis. * p≤0.05, ** p≤0.01, *** p≤0.001.

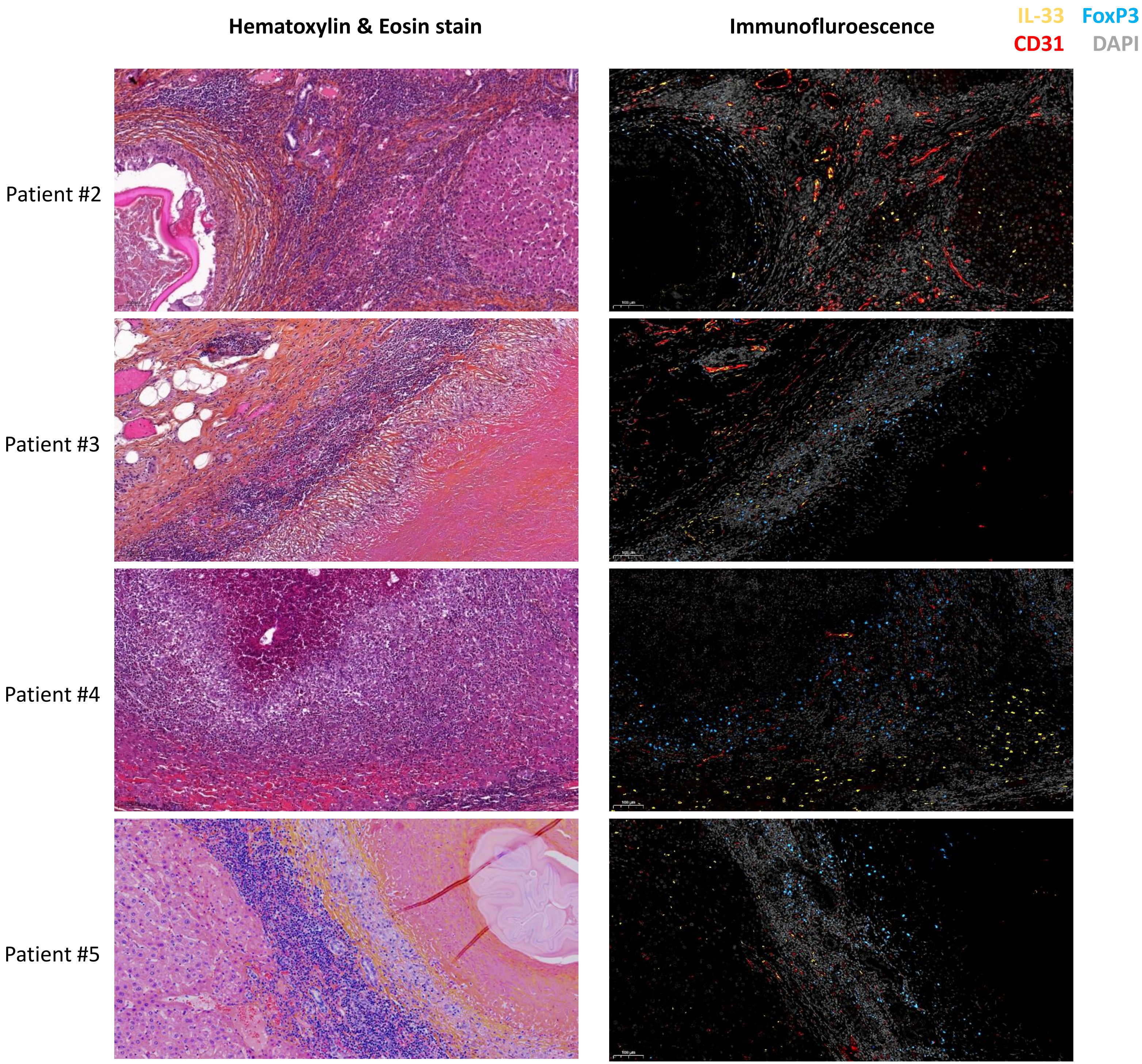

Supplementary Figure 3. Immunofluorescence staining of human alveolar echinococcosis biopsies. Hematoxylin and eosin staining and immunofluorescence staining of consecutive histological sections, using DAPI, anti-IL-33, anti-CD31, and anti-FoxP3 antibodies, for 4 patients with alveolar echinococcosis.

Supplementary Table 1. List of staining reagents used for flow cytometry

**Antibodies/viability stain**	**Provider**	**Comments**
**Panel 1**		
Anti-CD19-BV786 (1D3)	BD Biosciences, Le Pont de Claix, France	
Anti-CD3-APC (145.2.C11)	BD Biosciences, Le Pont de Claix, France	
Anti-NK1.1-PE/CF594 (PK136)	BD Biosciences, Le Pont de Claix, France	
Anti-Gr1-BV421 (RB6-8C5)	BD Biosciences, Le Pont de Claix, France	
Anti-CD11b-PE/Cy7 (M1/70)	BD Biosciences, Le Pont de Claix, France	
Anti-CD8-APC/Cy7 (53-6-7)	BD Biosciences, Le Pont de Claix, France	
Anti-CD4-BV510 (RM 4-5)	BD Biosciences, Le Pont de Claix, France	
Anti-FoxP3-PE (150D/E4)	eBioscience™, Thermo Fisher Scientific®, Illkirch, France	
LIVE/DEAD™ Fixable Green Dead Cell Stain	Invitrogen™, Thermo Fisher Scientific®, Illkirch, France	
**Panel 2**		
Anti-Siglec-F-PE/Dazzle594 (S17007L)	Biolegend®, Paris, France	Included in 1 experiment replicate
Anti-CD11c-PE/Cy7 (N418)	Biolegend®, Paris, France	
Anti-CD11b-BV510 (M1/70)	BD Biosciences, Le Pont de Claix, France	
Anti-CD206-PE (C068C2)	Biolegend®, Paris, France	
Anti-I-A/I-B (MHC-II)-FITC (M5/114.15.2)	Biolegend®, Paris, France	
Anti-CD3-PerCP/Cy5.5 (145-2C11)	Biolegend®, Paris, France	Part of “Dump”
Anti-CD19-PerCP/Cy5.5 (6D5)	Biolegend®, Paris, France	Part of “Dump”

Anti-NK1.1-PerCP/Cy5.5 (PK136)	Biolegend®, Paris, France	Part of “Dump”
Anti-CD64-APC (X54-5/7.1)	Biolegend®, Paris, France	
Anti-Ly6C-BV711 (HK1.4)	Biolegend®, Paris, France	
Anti-Ly6G-APC/AF700 (1A8)	Biolegend®, Paris, France	Included in 1 experiment replicate
Zombie NIR Fixable Viability Kit	Biolegend®, Paris, France	

Supplementary Table 2. List of used primers

Targeted gene	Forward primer sequence	Reverse primer sequence
Actb	CTG TCC CTG TAT GCC TCT G	ATG TCA CGC ACG ATT TCC
Gapdh	TGC ACC ACC AAC TGC TTA GC	GCA TGG ACT GTG GTC ATG AG
Ppid	ATG GTC AAA AAC CTG CCA AA	CAT CCT CAG GGA AGT CTG GA
Il1rn	GAA AAG ACC CTG CAA GAT GC	GCC CAA GAA CAC ACT ATG AAG G
Il1b	GAT CCA CAC TCT CCA GCT GCA	CAA CCA ACA AGT GAT ATT CTC CA
Tgfb1	CAC CAT CCA TGA CAT GAA CC	CAG AAG TTG GCA TGG TAG CC
Tnfa	TAG CTC CCA GAA AAG CAA GC	TTT TCT GGA GGG AGA TGT GG
Il17f	TGC CAT TCT GAG GGA GGT AG	ACA GAA ATG CCC TGG TTT TG
Il10	GGT TGC CAA GCC TTA TCG GA	ACC TGC TCC ACT GCC TTG CT
Il12p35	CAG CAG CTC CTC TCA GTG C	GTG GTC TTC AGC AGG TTT CG
Il6	CCG GAG AGG AGA CTT CAC AG	TCC ACG ATT TCC CAG AGA AC
Il4	GGC TTC CAA GGT GCT TCG	GGA CTT GGA CTC ATT CAT GG
Ifng	AGG TCA ACA ACC CAC AGG TC	ATC AGC AGC GAC TCC TTT TC
Arg1	CTC CAA GCC AAA GTC CTT AGA G	GGA GCT GTC ATT AGG GAC ATC A
Csf2	GGC CTT GGA AGC ATG TAG AGG	GGA GAA CTC GTT AGA GAC GAC TT
Cd163	GGT GGA CAC AGA ATG GTT CTT C	CCA GGA GCG TTA GTG ACA GC
Tlr8	TTC CAG AAG CTA TCC TTG TGA CG	CAT GCA GTT GAC GAT GGT TGC
Em14-3-3	GAT AGT ACT CTC ATC ATG CAG	CTC AAT CAG AAC CAC GAC AG
ActII	CGC GAT CTC ACC GAC TGG	CTC CAG AGA GGA GCT AGT G
